# Motion onset VEPs can see through the blur

**DOI:** 10.1038/s41598-024-72483-z

**Published:** 2024-09-12

**Authors:** D. Kordek, L. Young, P. Voda, J. Kremláček

**Affiliations:** 1 Department of Medical Biophysics, Faculty of Medicine in Hradec Kralove, Charles University, Simkova 870, 500 03 Hradec Kralove, Czechia; 2 Faculty of Medical Sciences, Biosciences Institute, Newcastle University, Newcastle upon Tyne, UK

**Keywords:** Pattern-reversal, Motion-onset, Digital blur, Visual evoked potential, Neuroscience, Visual system

## Abstract

Motion-onset visual evoked potentials (MO VEPs) are robust to dioptric blur when low contrast and low spatial frequency patterns are used for stimulation. To reveal mechanisms of MO VEPs robustness, we studied whether the resistance to defocus persists even when using a high-contrast checkerboard using digital defocus in the emmetropic eyes of 13 subjects (males 20–60 years). We compared the dominant components of MO VEPs to pattern-reversal VEPs (PR VEP), which are sensitive to the blur. For stimulation, we used checkerboard patterns with 15´ and 60´ checks. To defocus the checkerboard, we rendered it with a second-order Zernike polynomial (${Z}_{2}^{0}$) with an equivalent defocus of 0, 2, or 4 D. For PR VEP, the checkerboards were reversed in terms of their contrast. To evoke MO VEP, the checkerboard of 60´ checks moved for 200 ms with a speed of 5 or 10 deg/s in the cardinal directions. The MO VEP did not change in peak time (P ≥ 0.0747) or interpeak amplitude (P > 0.0772) with digital blur. In contrast, for PR VEP, the results showed a decrease in interpeak amplitude (P ≤ 6.65ˑ10-4) and an increase in peak time (P ≤ 0.0385). Thus, we demonstrated that MO VEPs evoked by checkerboard, structure containing high spatial content, can be robust to defocus.

## Introduction

Visual evoked potentials (VEPs) allow testing of the integrity of the visual system objectively, non-invasively and with minimal material cost and thus they contribute to the eventual diagnosis of retrobulbar pathological processes. The basic diagnostic parameters of VEP are influenced by the luminance, and spatial and temporal properties of the stimulus. For optimal inter-laboratory interpretability and high diagnostic yield, it is a good idea to follow ISCEV standards^1^. When a stimulus is projected onto the retina, light passes through the optical media of the eye, so the image may be modified even at this early level and cause erroneous conclusions about retrobulbar pathology. For this reason, ISCEV standards recommend optimally correcting the refractive error of the eye for the stimulus distance and recording the refractive error in the examination report.

In some patients, correcting the eye's refractive error is difficult. For this reason, knowing how the VEP parameters change in response to refractive error, tested using artificially blurred stimuli in healthy subjects, can be helpful. Such knowledge might allow differentiation between normal or pathological states of the visual system in a patient with an uncorrected refractive error^2–4^.

Many studies have looked specifically at the effect of artificially blurred pattern stimuli on the fundamental characteristics of VEP (VEP specifically induced by checkerboard reversal—PR VEP). In these studies, a high degree of dependence of key VEP parameters on the spatial frequency of the stimuli has been demonstrated. Specifically, it has been shown that with increased blurring of the images (due to refractive errors), there is a significant increase in the peak time of the P100 component and a decrease in its amplitude^5–7^. In their early work, White and Harter (1968) demonstrated that blur systematically alters VEPs and patented VEP-based method to assess visual refractive error^8^ (for review see^9^).

The low specificity of the PR VEP limits the ability to separate the decrease in visual acuity caused by refractive error from retrobulbar impairment. For these reasons, VEP variants that have low sensitivity for refractive errors would be useful in patients with reduced visual acuity. Examples of such VEP examinations are motion-onset VEPs (MO VEPs), which can also be elicited in the periphery of the visual field^10–12^ by stimulating patterns with low spatial frequencies. The dependence of the characteristics of MO VEPs on artificially (dioptrically) induced refractive error was investigated by Kordek et al. 2022^7^. It was observed that dioptric blur significantly prolongs peak times and reduces interpeak amplitudes of PR VEPs, but not of MO VEPs. The experiment used clinically employed patterns that were optimized for activation of different visual channels. While a high-contrast checkerboard pattern with a significant representation of higher spatial frequencies was used for reversal stimulation, motion stimulation was elicited by a pattern with low content of higher spatial frequencies and low luminance contrast. The dissimilarity of the two patterns could also be a factor affecting the sensitivity of VEP to blur. For this reason, we performed another experiment in which we kept the structure identical for reversal and motion stimulation. At the same time, we used a digital blur, which allows for precise and consistent control of the blur parameters homogeneously throughout the stimulation area. It is also less prone to potential confounding factors related to lens-induced magnification or minification effects, vertex distance variation, and an imperfect optical axis alignment compared to dioptric blur^13,14^.

The aim of our study was to describe and compare the behaviour of the basic parameters of the PR VEP and MO VEP as a function of digitally induced second-order aberration (“defocus”) in the emmetropic eye and to answer the question of whether the robustness of the motion stimulus to blur persists when using a high-contrast checkerboard.

## Methods

### Subjects

A total of 13 men between the ages of 20 and 60 years (mean age 39 years) were examined. The subjects reported no neurological or ophthalmological problems at the time of examination. All subjects signed an informed consent and GDPR consent before the examination. All procedures performed in our study were in accordance with the ethical standards of the institutional and/or national research committee and with the 1964 Declaration of Helsinki and its later amendments or comparable ethical standards. The study was approved by the ethics committee of the University Hospital in Hradec Kralove (no. 201411S19P).

### Stimulation

VEP examination was performed in the neurophysiology laboratory of the Department of Pathological Physiology, Faculty of Medicine in Hradec Kralove, on a CRT monitor (Vision Master Pro 510, Iiyama, Japan) with a resolution of 1024 × 768 pixels and an angular size of 37° × 28°, from a viewing distance of 60 cm. The visual stimuli were generated using PsychoPy v2022.2.5 (Open Science Tools Ltd., UK). For the examination of PR VEP and MO VEP, checkerboards of 15´ and 60´ square angular size were used as patterns according to ISCEV standards^1^. Each stimulation included the introductory text “Get ready, the stimulation is about to begin”.

During the PR VEP (simple checkerboard reversal) examination, the Michelson contrast between the white and black squares was 96%. The mean luminance was 17 cd/m^2^, constant throughout the experiment. The checkerboard was displayed in the full monitor area and reversed twice within one second. A red fixation mark was displayed in the centre of the monitor throughout. Each PR VEP examination (PR 15´, PR 60´) lasted approximately 25 s and consisted of 50 stimuli.

The MO VEP examination in this study was performed with the checkerboard of 60´ square angular size, with two temporal frequency values of 2 Hz (MO TF2) and 4 Hz (MO TF4). We choose to move the larger checkerboard only to keep stimulus parameters optimal for MO VEPs (size of the structure between 0.2 and 1.0 c/deg, and speed from 5 to 25 deg/s)^10^. For the checkerboard of 15´ square angular size, we would need a slow motion of 1 deg/s to keep the same temporal frequency (2 Hz) as for the larger checks. For such speed, it is necessary to move the pattern by 0.5 pixel/frame, which interrupts the smoothness of the motion in our setup.

**Fig. 1:**
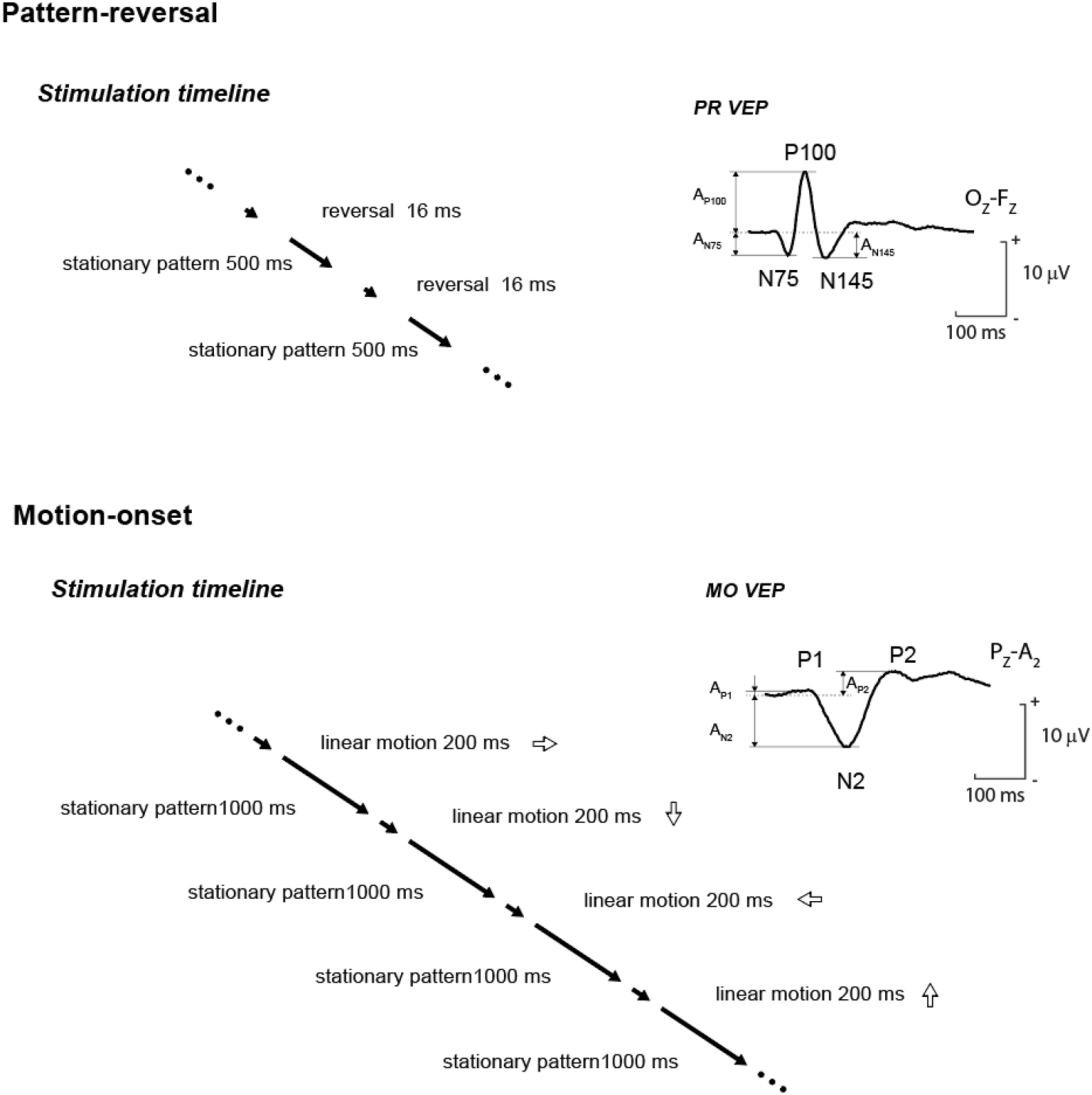
Schematic of the changes for the reversal (top) and motion stimuli (bottom), including a typical VEP waveform in the right part of the figure, where the evaluated parameters of the dominant peaks are marked.

Michelson contrast between white and black squares was 96%. Mean luminance was 17 cd/m^2^, constant throughout the experiment. A red fixation mark was displayed in the centre of the monitor throughout. The motion and reversal pattern schemes are shown in Fig. 1.

VEPs were recorded using unipolar electrodes (O_Z_, P_Z_, C_Z_, F_Z_ and O_L_, O_R_—5 cm to the left and right of O_Z_). The reference electrode was placed in position A_2_, the ground electrode was on the left wrist. EEG recording was performed on a TruTrace device (Deymed Diagnostic s.r.o., Czech Republic) with a sampling frequency of 6250 Hz and a frequency bandwidth of 1—1000 Hz. The signal was then digitally filtered in the band 1—40 Hz using the pop_eegfiltnew function^15^. For display, the signal was smoothed with a Savitzky-Golay filter with a window width of 50 ms and 1^st^ order interpolation (sgolayfilt.m, Matlab rel. 2023a, Mathworks, Inc., USA).

### Digital blur

In our study, the simulation of second-order aberration (“defocus”—${Z}_{2}^{0}$) was induced as described in a study by Kordek et al. 2021^16^. Thus, the resulting defocused checkerboards (of both angular sizes) were produced by computer convolution of the defocused checkerboards and the point spread function (PSF) described in^14,17,18^, which for the case of ${Z}_{2}^{0}$ aberration is given by Eq. (1)^16^,

1 $$PSF\left( {r,\theta } \right) = \left| { \cdot \left\{ {P\left( {r,\theta } \right)} \right\}} \right|^{2} = \left| { \cdot \left\{ {p\left( {r,\theta } \right) \cdot \exp \left( {i \cdot \frac{2\pi }{\lambda } \cdot RMS \cdot \sqrt 3 \cdot z \cdot \left( {2 \cdot \rho^{2} - 1} \right)} \right)} \right\}} \right|^{2}$$

The PSF is the squared modulus of the Fourier transform of the complex aperture function. The function $$p\left(r,\theta \right)$$ represents the aperture’s amplitude function and has in our case is circular with a value of 1 inside the pupil and a value of 0 outside the pupil. The expression $$\sqrt{3}\cdot \left(2\cdot {\rho }^{2}-1\right)$$ corresponds to the second order Zernike polynomial $${Z}_{2}^{0}$$^19^,where ρ is the radial distance in the pupil. This is scaled by $${\raise0.7ex\hbox{${2\pi }$} \!\mathord{\left/ {\vphantom {{2\pi } \lambda }}\right.\kern-0pt} \!\lower0.7ex\hbox{$\lambda $}}^{\prime}$$, where λ is the wavelength of light (570 nm), and by the RMS Zernike coefficient to give the phase of the wavefront. The RMS Zernike coefficient is an optical metric that determines the degree of blurring of the checkerboard, defined as the root-mean-square of the wavefront ^17^. In our case, the RMS takes values of 0, 1.8 and 3.6 µm, which corresponds to equivalent defocus values, *M*_*e*_, of 0, 2 and 4 D in turn, based on the Eq. (2)^20^

2 $$M_{e} = 4 \cdot \pi \cdot \sqrt 3 \cdot \frac{RMS}{A} = \frac{4 \cdot \sqrt 3 \cdot RMS}{{R^{2} }}$$

where *R* is the pupil radius. For the correct construction of the transformed image, the pixel scale of the PSF (*s*_*PSF*_) must be equal to the pixel scale of the source image (*s*_*obj*_), i.e. *s*_*PSF*_ = *s*_*obj*_. We started from the Fraunhofer diffraction theory for a rectangular aperture, where^18^ *s*_*PSF*_ = λ / *D*∙α.

where *D* is the aperture diameter of the aperture and α is the oversampling factor^18^. The pixel size of the source image is given by *s*_*obj*_ = *v*_*obj*_ / *N*_*obj*_, where *N*_*obj*_ is the number of pixels in the field of view of the source image and *v*_*obj*_ is the field of view of the source image. Comparing the two scales, we obtain the following equality ”Eq. (3)”^18^,

3 $$\frac{\lambda }{D \cdot \alpha } = \frac{{v_{OBJ} }}{{N_{OBJ} }} \Rightarrow N_{OBJ} = \frac{{v_{OBJ} \cdot D \cdot \alpha }}{\lambda }$$

Based on this theory, custom code for blurring the checkerboard was created, adapted from Young & Smithson (2014)^18^, in the Python programming language using the NumPy library^21^. The parameters of the source code were designed for a viewing distance of 60 cm and an oversampling factor of 2 was used. The resulting blurred checkerboards were generated at a resolution of 1024 × 768 pixels. To allow a motion animation, the checkerboards were extended to fill a canvas size of 2017 × 2015 pixels.

**Fig. 2:**
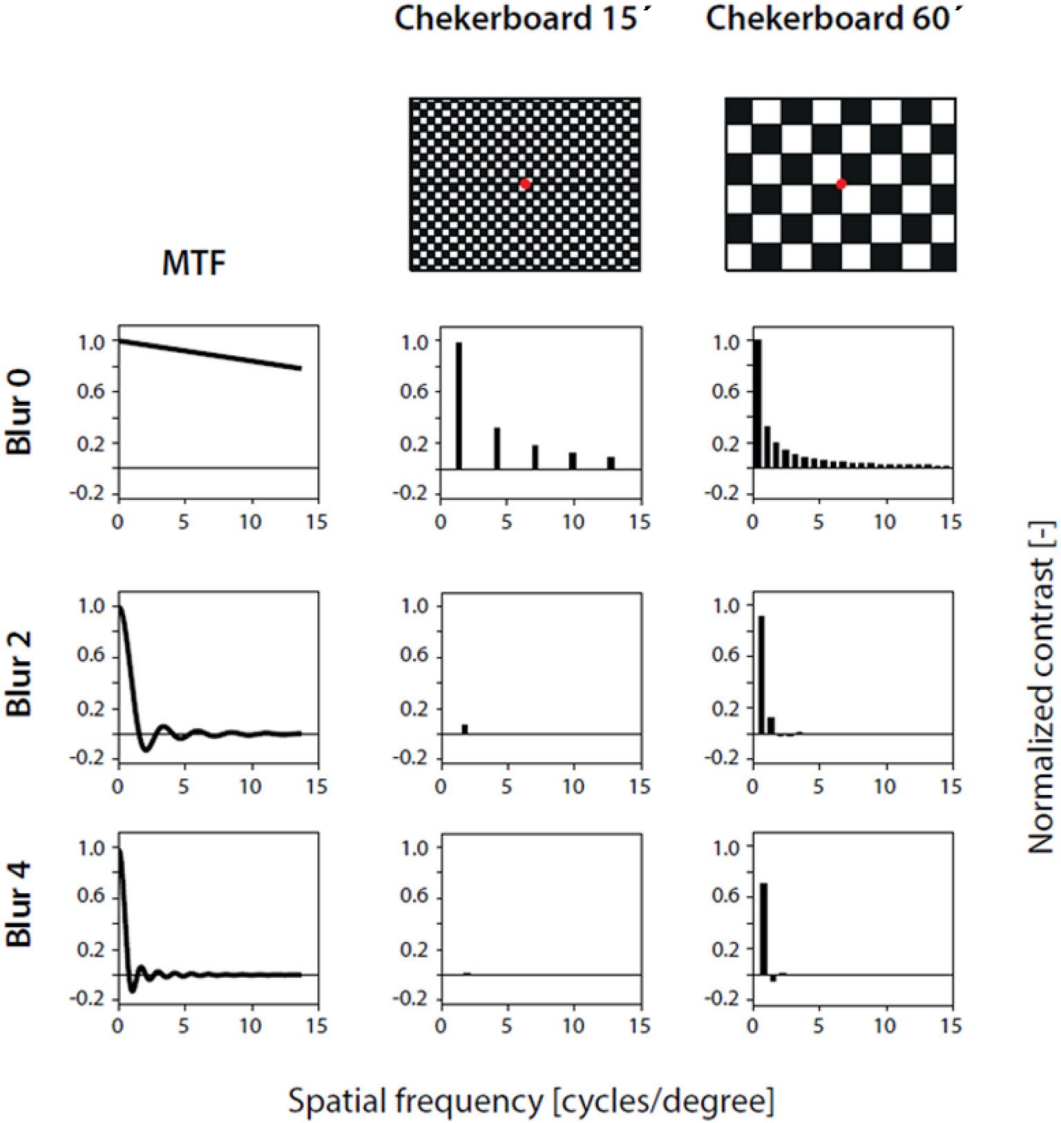
Representation of the transfer function of the filter (first column—MTF) that was used for digital blur of the checkerboard patterns. The original patterns are shown symbolically in the first row of the second and third columns. Rows 2, 3 and 4 indicate the digital blur corresponding to 0 D (Blur 0), 2 D (Blur 2) and 4 D (Blur 4), respectively. The spectral content of the filtered patterns in the second and third columns was calculated analytically as the product of the transfer function with the one-dimensional spectral brightness profile obtained along the diagonal of the checkerboard (rectangular waveform). The spectral content plots show that the 15´ checkerboard was most affected at Blur 4. The actual stimulus pattern and degradation are available in the supplementary material “digital_blur_stimuli.zip”.

The checkerboards for each examination were digitally blurred with equivalent defocus values of 0, 2 and 4 D (denoted “Blur 0”, “Blur 2” and “Blur 4”, respectively). The transfer functions for each blur value and the frequency spectra of the checkerboards are shown in Fig. 2, where only symbolic representations of the patterns before blurring are shown. Patterns at the stimulus screen resolution for all blur levels are available in the supplementary material “digital_blur_stimuli.zip”.

### VEP recording

First, we measured the refractive state of both eyes of the subject using a Nidek Ark1a auto refractometer and the better eye was selected for the following examination. In the case of equal refractive status of both eyes, we selected the so-called dominant eye using the Hole-in-Card Test (Dolman's method)^22,23^. Then, in the first step, visual acuity was measured monocularly using Landolt C optotypes^24^ (viewing distance 4 m), and in the second step, contrast sensitivity was examined using CSV-1000 E^25,26^ (viewing distance 2.5 m, total luminance 85 cd/m^2^, VectorVision, Guardion Health Sciences Inc., USA).

The VEP examination itself was also performed monocularly. For each subject, we performed the four aforementioned VEP stimulations (PR 15´, PR 60´, MO TF2, MO TF4). For each subject, the examination was performed twice, giving two repeated measurements for each equivalent defocus value and for each VEP stimulation. For each stimulation and blur, the resulting record was constructed as an average of both repetitions. Thus, 12 VEP records were obtained for each subject. For each person, we randomised the order of the stimulations and the blurring levels to eliminate the effect of adaptation and fatigue^27,28^. For all subjects, to remove additional effects from their refractive error we always added a visual correction measured with an auto refractometer using the dioptric test set Art. 51-BL, M.S.D. in front of the eye under examination, analogous to the methodology used in our previous study^16^.

### Statistical analysis

For the PR VEPs, the peak time and amplitude values of N75, N145, and P100 components were determined from the O_Z_-F_Z_ lead. For the MO VEPs, the peak time and amplitude values for P1, N2, and P2 were read from the PZ-A2 lead. Data evaluation was performed by authors DK and JK.

Peak times P100 and N2 and interpeak amplitudes PR-Am (A_P100 – (A_N75 + A_N145)/2) and, MO-Am ((A_P1 + A_P2)/2 – A_N2) were used for subsequent analysis, a methodology analogous to our previous study^7^.

The relationship between the aforementioned parameters and the blur (i.e., the equivalent defocus value), was described for each subject and VEP stimulus using a linear regression model. This resulted in regression line slopes for all subjects for the 4 stimulations and two parameters (peak time and interpeak amplitude). The normality of each set was tested by the Shapiro–Wilk test. We tested whether the slopes differed from zero by Student's t-test or Wilcoxon test (when normality was not met). Data processing was performed with the Jamovi program (The jamovi project (2023). Jamovi (Version 2.3) [Computer Software]. Retrieved from https://www.jamovi.org). Use of a different regression model, such as a logarithmic regression model, might generally not yield statistically significantly different results. Evidence for this claim is, in our study, the very small difference in the coefficient of determination for the two regression models. In this case, it is customary to choose the simpler, i.e., linear model. This model was also used by Bobak et al. (1987)^29^.

## Results

The measured visual acuity of the subjects ranged from 0.8 to 1.6 (nine out of thirteen subjects have a VA value of 1) in decimal expression. Each subject was assigned their necessary dioptric correction in front of the eye and these ranged from − 0.5 D to 0.5 D (median = 0.25 D, Q1 =  − 0.25 D, Q3 = 0.25 D).

**Fig. 3:**
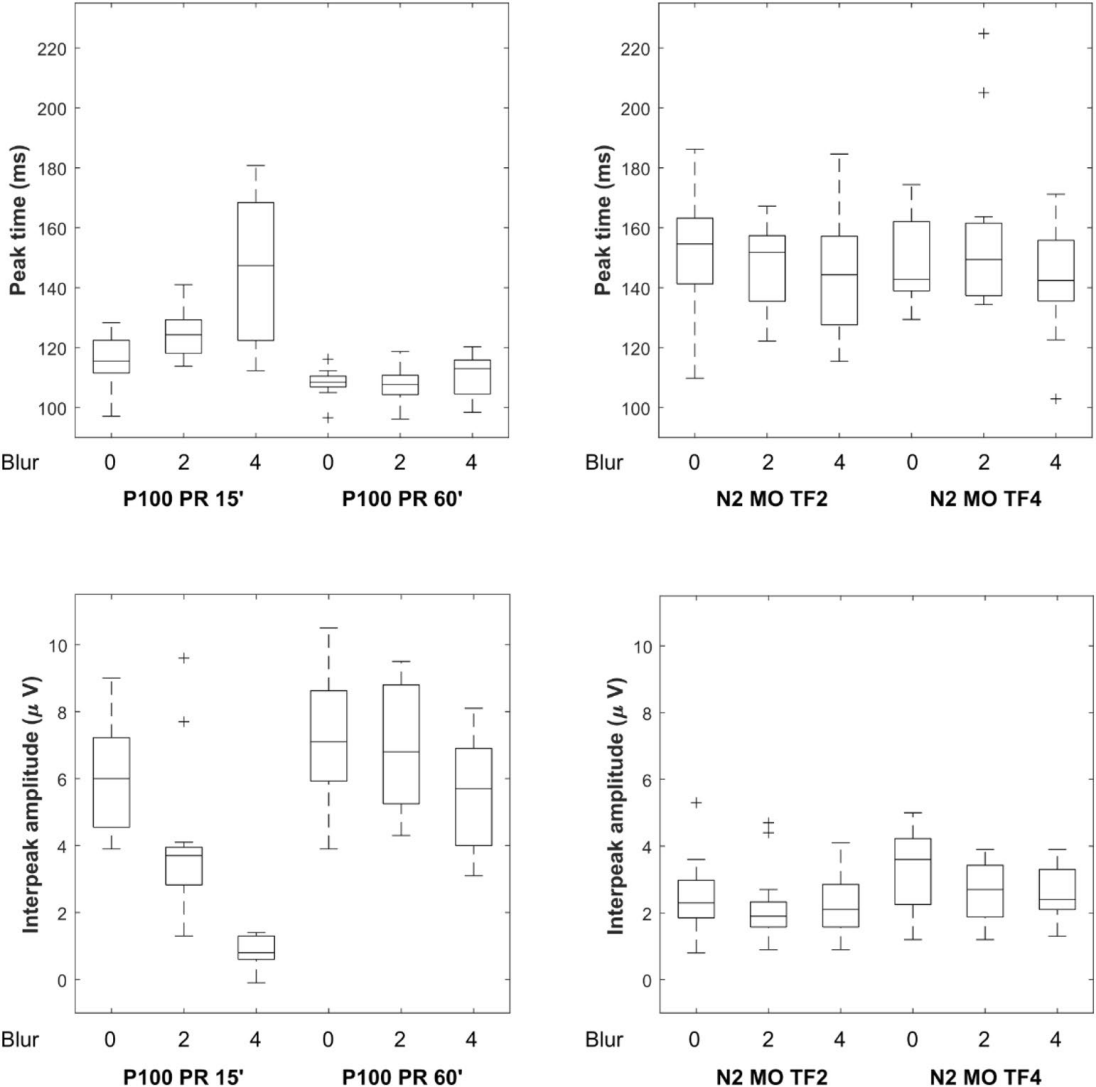
Graphical representation of the distribution of the interpeak amplitude and the peak time values for the PR VEP and MO VEP parameters. The upper graphs show the values for peak time and the lower graphs show the values for interpeak amplitude. Box plots indicate the median, upper and lower quartiles, dashed lines indicate the 25th and 75th percentiles, and the + sign indicates outliers.

**Fig. 4:**
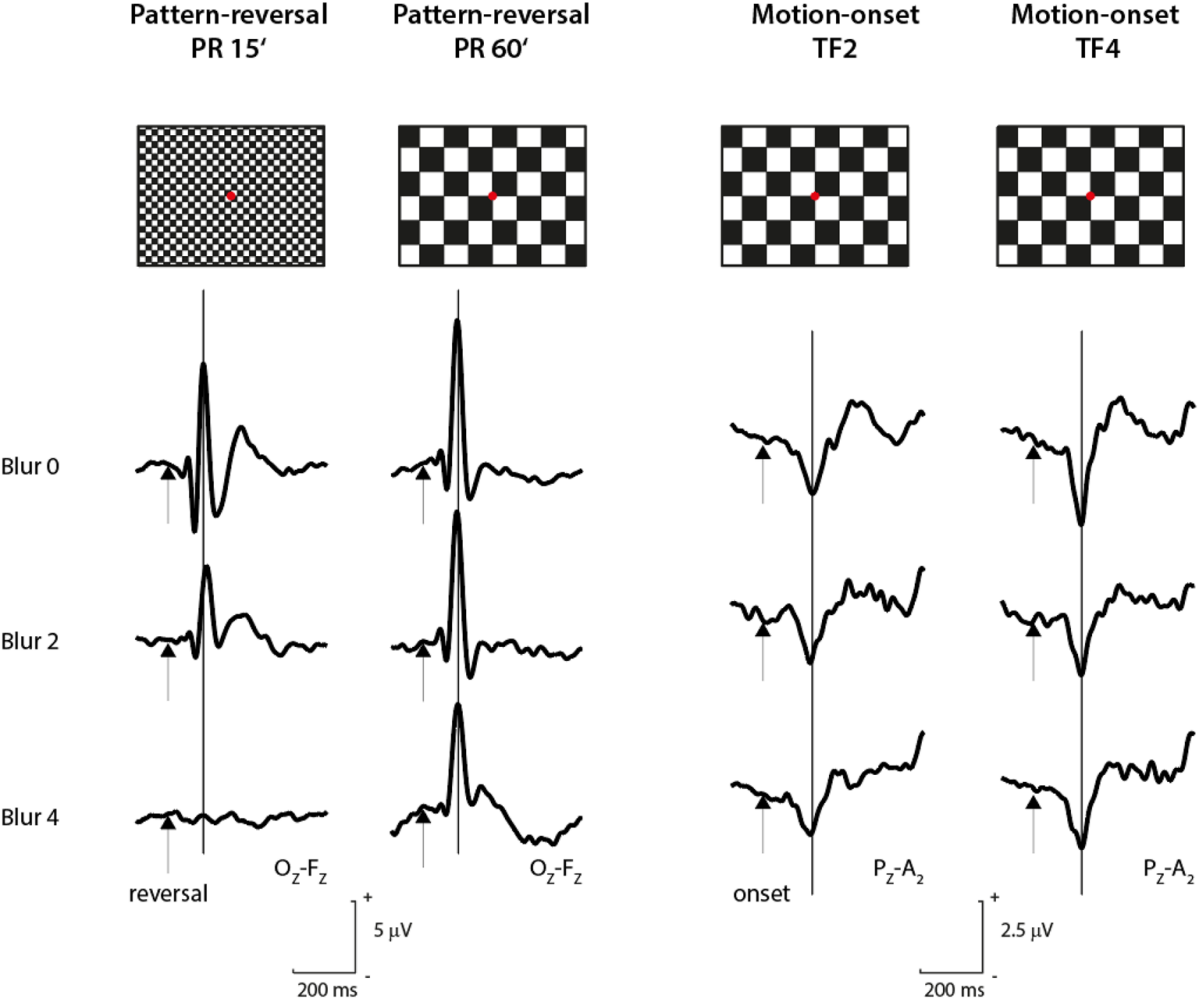
Grand average VEP recordings across subjects for the four stimulations and three blur conditions used. At the top of the figure, icons show the specific stimulus pattern: from the left are the stimulations PR 15´, PR 60´, MO TF2, and MO TF4. In the bottom part, the VEP grand averages over all subjects for a particular stimulation and digital blur condition are depicted. The rows show the effect of blurring. The changes in the first two columns correspond to the sensitivity of the PR VEP to digital blur. In contrast, the stability of the responses in the right two columns illustrates the robustness of the MO VEP.

Basic descriptive statistics of peak times and interpeak amplitudes for VEP stimulations and blur conditions are shown in Table 1 and Fig. 3. The VEP grand averages are shown in Fig. 4. Source values from all subjects used to calculate the values in Table 1 are available in the supplementary material (Table S1 and Table S2).

**Table 1:** Median, 25th and 75th percentile peak times and interpeak amplitudes. Values represent the median and Q1 and Q3 calculated across all subjects for each stimulation and blur.

Blur condition		P100 PR 15’	P100 PR 60’	N2 MO TF2	N2 MO TF4
Blur 0	Peak time [ms]	115.5 (111.8, 122.2)	108.5 (107.0, 110.2)	154.6 (141.8, 162.9)	142.7 (139.2, 161.6)
Blur 2		124.3 (118.2, 126.9)	107.7 (104.8, 110.7)	151.8 (135.5, 156.2)	149.4 (137.6, 160.8)
Blur 4		147.4 (124.2, 164.0)	113.0 (104.8, 115.7)	144.3 (129.0, 156.6)	142.4 (136.8, 155.2)
		PR-A_m_ PR 15’	PR-A_m_ PR 60’	MO-A_m_ MO TF2	MO-A_m_ MO TF4
Blur 0	Interpeak Amplitude [µV]	6.0 (4.6, 7.1)	7.1 (6.1, 8.5)	2.3 (1.9, 2.8)	3.6 (2.3, 4.1)
Blur 2		3.7 (3.1, 3.9)	6.8 (5.5, 8.7)	1.9 (1.6, 2.2)	2.7 (1.9, 3.4)
Blur 4		0.8 (0.6, 1.3)	5.7 (4.2, 6.9)	2.1 (1.6, 2.8)	2.4 (2.1, 3.2)

**Fig. 5:**
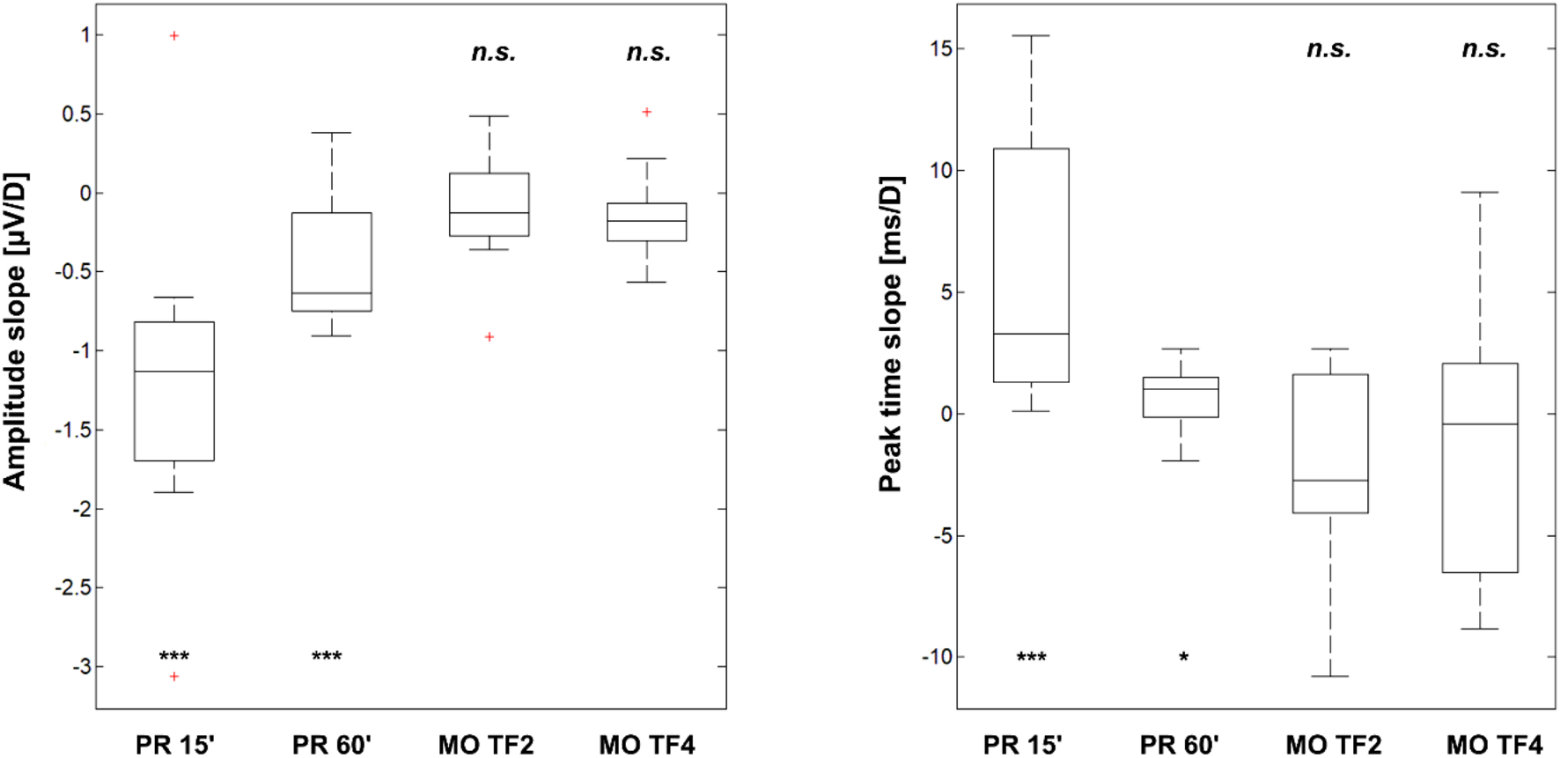
Graphical representation of the distribution of the regression line slopes for the PR VEP and MO VEP parameters. The directive describes the estimate of how the corresponding parameter changes when the equivalent defocus is increased by 1 D. The left plot shows the slopes for the interpeak amplitude, and the right plot shows the slopes for the peak time. Box plots indicate the median, upper and lower quartiles, dashed lines indicate the 25th and 75th percentiles, and the + sign indicates outliers. Above or below every boxplot is indicated if the slope was significantly different from zero (*p < 0.05, ***p < 0.001, n.s. indicates not significant).

The basic descriptive characteristics of the linear trends and the error probability of rejecting the true null hypothesis in the regression analysis are summarized in Table 2 and Fig. 5.

**Table 2:** Basic descriptive statistics of the regression line slopes peak time P100 and interpeak amplitude PR-Am for PR 15´ and PR 60´ stimulations and peak time N2 and interpeak amplitude MO-Am for MO TF2 and MO TF4 stimulations.

		PR 15’	PR 60’	MO TF2	MO TF4
Peak time	median, Q1, Q3 [ms/D] *p*-value	3.28, 1.35, 10.4 1.22 × 10^−4^*	1.00, 0.075, 1.30 0.039	− 2.73, − 4.08, 1.56 0.075	− 0.4, − 6.04, 1.88 0.33
Interpeak amplitude	median, Q1, Q3 [µV/D] *p*-value	− 1.13, − 1.69, − 0.85 2.95 × 10^−4^	− 0.64, − 0.74, − 0.13 6.65 × 10^−4^	− 0.13, − 0.26, 0.11 0.36	− 0.18, − 0.26, − 0.073 0.077

The “p-value” represents the p-value of the one-sample t-test (*respectively Wilcoxon test) of the arithmetic mean of the slopes of the regression lines of each parameter against zero.

This analysis shows that in the case of the PR VEP, there is a significant (*p* ≤ 6.65ˑ10^–4^) decrease in the PR-Am interpeak amplitude and a significant increase (*p* ≤ 0.0385) in the P100 peak time with increasing digital blur. For the PR 15´ (PR 60´) examination, the interpeak amplitude decreased by − 1.2 (− 0.5) µV and the dominant peak lengthened by 6 (1) ms with each dioptre of equivalent defocus of digital blur.

In the case of MO VEP, there was no significant (*p* ≥ 0.36, *p* ≥ 0.077) change in MO-Am interpeak amplitude or N2 peak time (*p* ≥ 0.075,* p* ≥ 0.33) with increasing digital blur in either the MO TF2 or MO TF4 condition.

The results of the regression analysis for all subjects, stimuli and parameters are presented in the supplementary material (Tables S3 and S4).

## Discussion

As early as the 1960s, image blur was recognized as an essential factor influencing VEPs. Responses to blurred patterns have been investigated, for example, with flashes illuminating a cardboard structure^30^, the presentation of slides^31^, or viewing on television or computer screens. These experiments were the basis for the objective selection of refractive correction^13,32,33^ and are now followed by an objective assessment of visual acuity^34^. Investigating the effect of defocus on VEPs is also an approach that can elucidate the genesis of VEPs in both normal and pathological situations and, because of the objective nature of the results, can be a helpful tool for verifying and calibrating models of visual perception.

In our study, we examined VEPs in response to two partially independent modalities of stimulation: pattern-reversal and motion onset. With the same spatial characteristics of the pattern used, the stimulations differed in the speed of the change required to elicit a VEP. For the local instantaneous contrast change in a pattern-reversal stimulus, as the digital defocus value increased, the interpeak amplitude of PR-Am decreased, and the peak time of P100 was prolonged. However, under motion onset stimulation where local contrast changed more slowly, the interpeak amplitude MO-Am and the peak time of N2 remained without statistically significant changes.

Similar published experiments for PR VEPs showed that changes in both parameters are more marked at the blur of a small structure in agreement with our results. However, they differ in the actual degree of amplitude decrease and peak time prolongation^7^,^35–38^. The variability in the results of these studies can be attributed to factors not controlled between studies, in particular, the method of image blurring, the magnitude of brightness and contrast of the stimulating structure used, or the population of subjects examined.

Only one study has been published for MO VEP, which is from our laboratory^7^. The current results agree with our prior findings, which is an important finding because the two studies have a significant methodological difference. In the first study, the stimulus pattern for motion-onset was a set of low-contrast (Michelson contrast 10%) concentric circles with reduced higher spatial frequencies (less than 1 c/deg). During the review process, a question arose as to whether a high-contrast structure, particularly one that is used for pattern-reversal VEP, could also be used for vision with refractive errors testing similar way as the low-contrast low spatial frequency structure. The presence of high spatial frequencies or high contrast may interfere with the resistance of the MO VEP to dioptric blur^7^. In the current study, we used a high-contrast checkerboard pattern (Michelson contrast 96%) with spatial frequencies exceeding 10 c/deg. However, the MO VEPs continue to show resistance to blur (despite the PR VEP defiance). We can conclude that neither the high contrast nor high spatial content of the stimulus pattern is critical in the resistance of the MO VEP to blur.

Considering the properties of the pattern-reversal and motion onset stimuli, we may conclude that the remaining and responsible difference for the blur resistance is the more gradual stimulus change (200 ms of relatively slow motion). The temporal tuning of the stimuli results in different neural processing of the motion onset and pattern-reversal^39,40^. While the dominant component of MO VEP peaks above the parietal area at about 160 ms (N2 peak), the P100 of the PR VEP culminates above the occipital cortex at about 100 ms. We showed that these neural processes exhibit different resistance to the blur.

Our results correspond to an expected effect of the second order aberration (“defocus” with a low pass transfer function; see Fig. 2) on the checkerboard. The low spatial frequencies perceived even after blurring are sufficient to evoke MO VEP but not PR VEP responses. Considering the properties of the pattern-reversal and motion onset stimuli, we may infer that the remaining and responsible difference for the blur resistance is the temporal change of the pattern. For MO VEP, the pattern changes position gradually for about 200 ms, allowing detection even of low contrast and low spatial frequency patterns. For PR VEP, the fast stimulus change (less than 14 ms – e.g. a video frame), makes the detection of degraded stimulus more difficult.

## Limitations

Our conclusion regarding the robustness of MO VEPs to defocus is based on the non-significant effects of blur observed in MO VEPs, in contrast to the significant effects seen in PR VEPs. It is important to consider that the lack of significant blur effects in MO VEPs may be due to higher noise levels or greater variability in MO VEP measurements compared to PR VEPs. This inherent noise could obscure the potential effects of blur on MO VEPs, leading to a non-significant result. From the variability listed in Table 1 and Fig. 3, such a possibility appears unlikely.

It should be noted that the different temporal properties of the stimuli result in different neural processing of the motion onset and pattern reversal stimuli^39,40^. While the dominant component of MO VEP peaks above the parietal area at about 160 ms (N2 peak), the P100 of the PR VEP culminates above the occipital cortex at about 100 ms. We showed that these neural processes exhibit different resistance to the blur. Our study only investigated specific velocities and spatial frequencies. Generalizability to other contexts might require further studies.

## Conclusion

We have shown that digital defocus of checkerboard stimuli up to an equivalent defocus value of 4 D does not cause a significant prolongation or reduction of the dominant component of motion onset VEPs, in contrast to pattern-reversal VEPs.

We conclude that for the temporal frequencies used, neither the high contrast nor high spatial content of the stimulus pattern interferes with the motion onset evoked potential’s resistance to blur. Therefore, if there is a need to study visual function in refractive error, response to the motion onset of a checkerboard can be a reliable method.

## Supplementary Information

Supplementary Information 1.

Supplementary Information 2.

Supplementary Information 3.

## Data Availability

The data we worked with in the analysis is available in the supplementary material of this paper.

## Code availability

The original code for rendering stimuli with Zernike defocus was released in the supplementary materials of Young & Smithson (2014)^16^.
