# Supplementary figures and images for: Motion onset VEPs can see through the blur
Source: Sci Rep. 2024 Sep 12;14:21296. doi: 10.1038/s41598-024-72483-z (PMC11393312; doi:10.1038/s41598-024-72483-z)

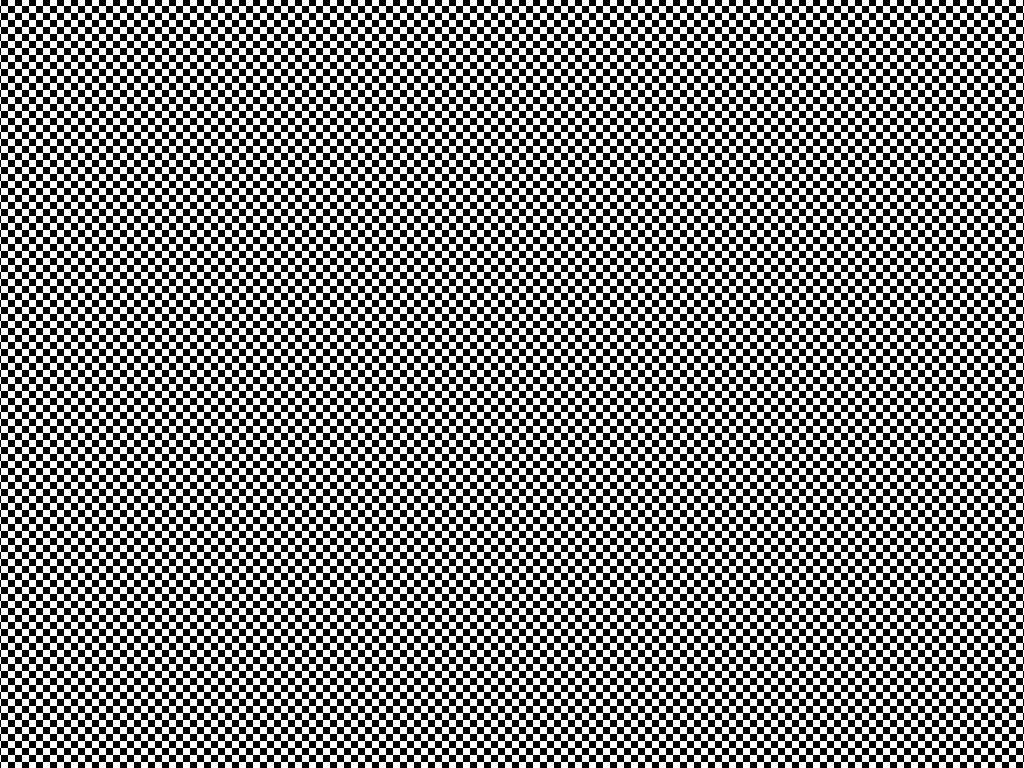

Supplement: Supplementary file 1 — Supplementary Information 1. [file 41598_2024_72483_MOESM1_ESM.zip › digital_blur_stimuli/appendix_digital_blur_VEP/Checkerboard_defocus_000_size_015.png]

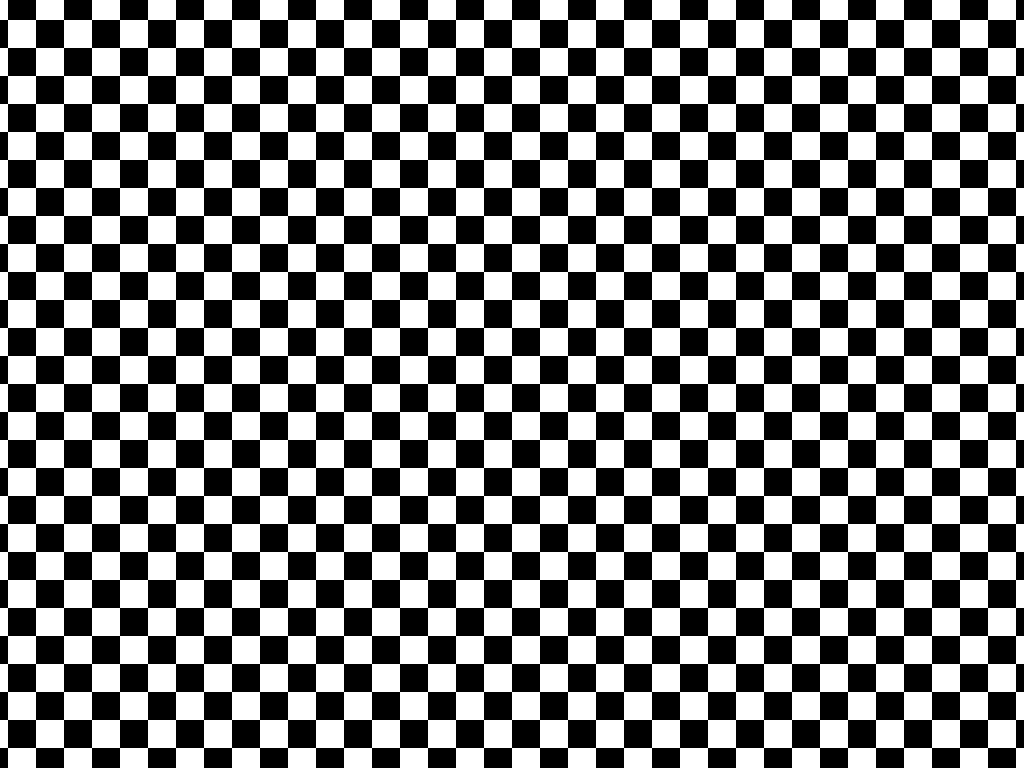

Supplement: Supplementary file 1 — Supplementary Information 1. [file 41598_2024_72483_MOESM1_ESM.zip › digital_blur_stimuli/appendix_digital_blur_VEP/Checkerboard_defocus_000_size_060.png]

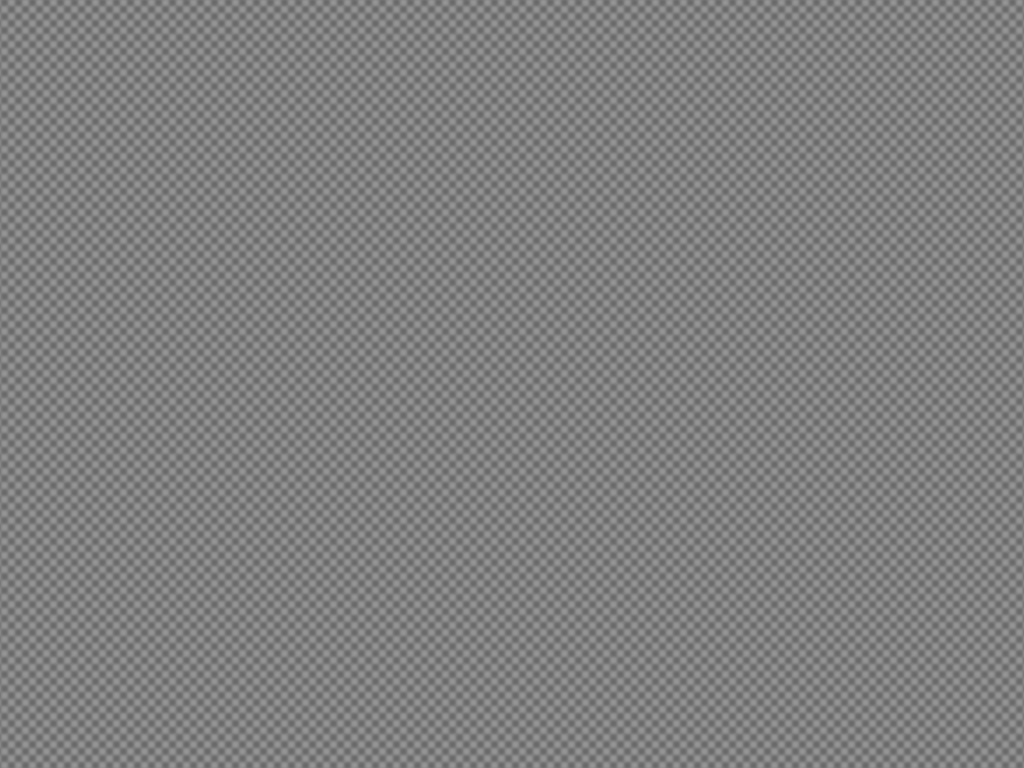

Supplement: Supplementary file 1 — Supplementary Information 1. [file 41598_2024_72483_MOESM1_ESM.zip › digital_blur_stimuli/appendix_digital_blur_VEP/Checkerboard_defocus_200_size_015.png]

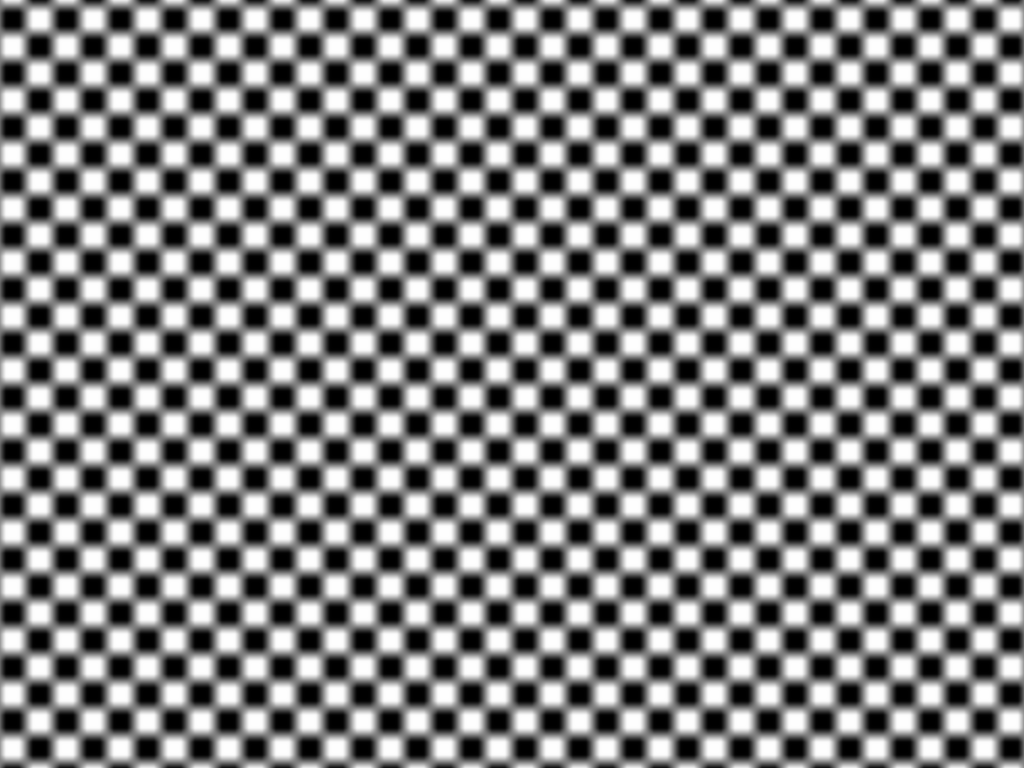

Supplement: Supplementary file 1 — Supplementary Information 1. [file 41598_2024_72483_MOESM1_ESM.zip › digital_blur_stimuli/appendix_digital_blur_VEP/Checkerboard_defocus_200_size_060.png]

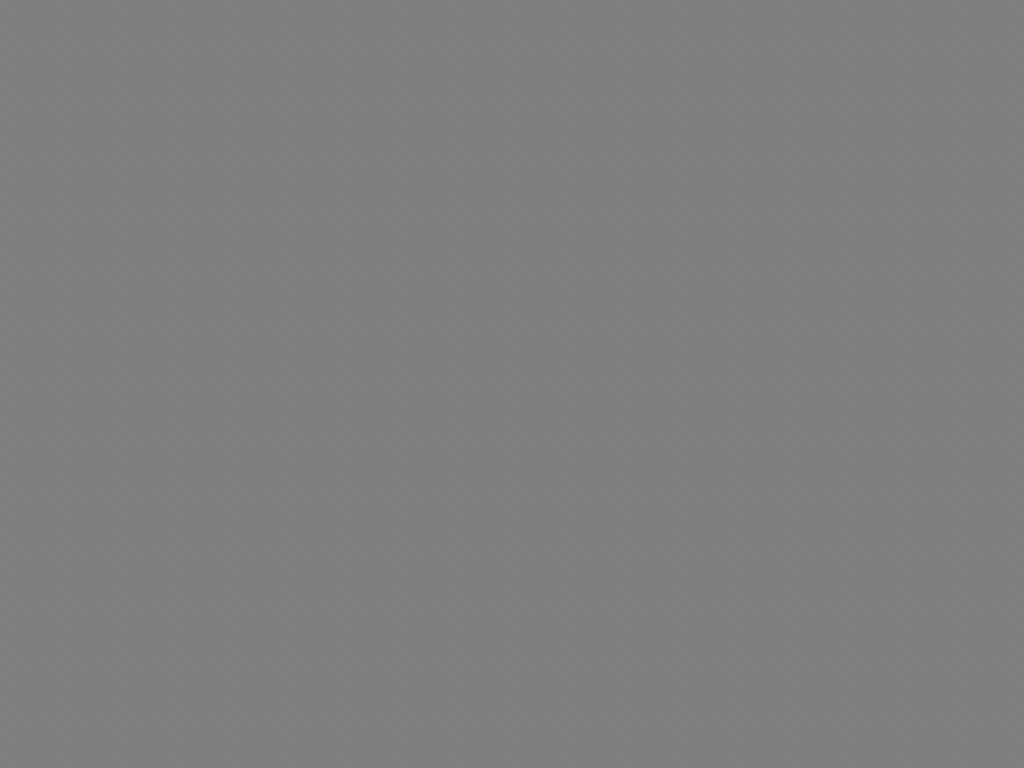

Supplement: Supplementary file 1 — Supplementary Information 1. [file 41598_2024_72483_MOESM1_ESM.zip › digital_blur_stimuli/appendix_digital_blur_VEP/Checkerboard_defocus_400_size_015.png]

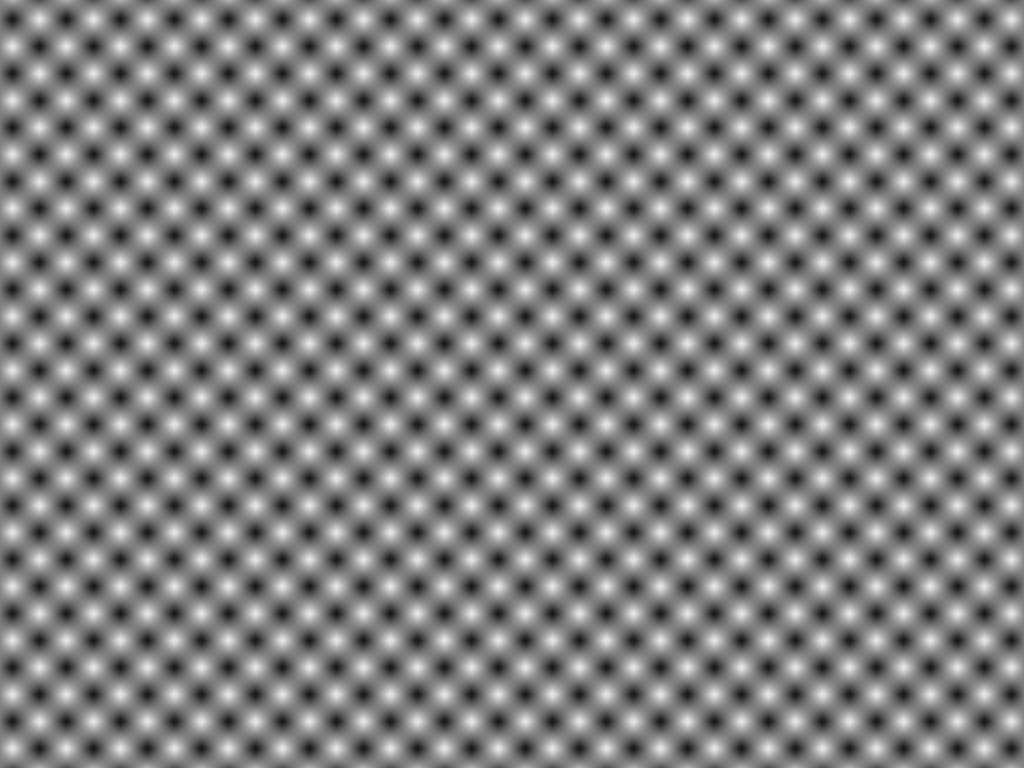

Supplement: Supplementary file 1 — Supplementary Information 1. [file 41598_2024_72483_MOESM1_ESM.zip › digital_blur_stimuli/appendix_digital_blur_VEP/Checkerboard_defocus_400_size_060.png]
