# Supplementary material for: Motion onset VEPs can see through the blur
Source: Sci Rep. 2024 Sep 12;14:21296. doi: 10.1038/s41598-024-72483-z (PMC11393312; doi:10.1038/s41598-024-72483-z)
Supplement: Supplementary file 2 — Supplementary Information 2. [file 41598_2024_72483_MOESM2_ESM.pdf]

Supplementary material

Motion onset VEPs can see through the blur.

D. Kordek[1], L. K. Young[2], P. Voda[1], J. Kremlacek[1]

[1]Department of Medical Biophysics. Faculty of Medicine in Hradec Kralove. Charles University. Hradec Kralove. Czech Republic

[2]Biosciences Institute. Newcastle University. Newcastle. UK

Table S1 – Peak time values [ms] for P100 positivity (for PR VEP) and N2 negativity (for MO VEP). Each value in the table is always the arithmetic mean of two measured values for each stimulation and for each digital blur value. Values in this table marked NaN are non-detectable values from PR 15´, Blur 4 records.

PR 15´			PR 60´			MO TF2			MO TF4		
0 D	2 D	4 D	0 D	2 D	4 D	0 D	2 D	4 D	0 D	2 D	4 D
P100			P100			N2			N2		
113.1	117.9	NaN	106.6	107.7	109.6	153.8	146.1	142.9	146.7	149.4	122.6
121.4	136.5	127.8	109.9	112.0	118.7	162.9	154.4	158.9	139.2	142.9	155.2
113.8	116.2	155.2	107.0	108.2	115.4	162.4	151.8	144.3	163.4	154.6	143.2
128.3	141.0	180.8	105.0	110.7	115.7	164.0	166.6	170.2	155.5	163.7	165.9
123.2	139.7	NaN	116.2	118.7	120.3	186.2	167.2	184.6	161.6	160.8	157.6
123.7	126.1	NaN	112.3	111.0	116.3	172.2	122.2	129.0	141.6	205.0	149.1
97.1	113.8	147.4	110.2	110.2	115.4	149.1	160.8	156.6	139.8	134.4	138.2
120.0	126.9	NaN	107.2	102.9	103.8	109.8	135.5	120.5	142.7	137.6	141.8
110.6	120.5	172.8	96.6	96.2	98.4	139.7	131.0	123.4	134.7	138.1	171.2
107.5	122.9	120.6	108.0	105.3	104.8	154.6	156.2	138.2	171.8	224.8	136.8
122.2	124.3	NaN	111.4	107.4	111.7	162.4	154.4	148.2	174.4	152.5	142.4
115.5	118.2	NaN	108.6	101.8	101.0	129.4	135.4	115.4	129.4	136.6	131.8
111.8	124.3	112.3	108.5	104.8	113.0	141.8	137.0	150.6	138.2	136.0	102.9

Table S2 – Interpeak amplitude values [μV] PR-Am = $(P100_{amplitude} - N75_{amplitude})/2 + (P100_{amplitude} - N145_{amplitude})/2$ and MO-Am = $(P1_{amplitude} - N2_{amplitude})/2 + (P2_{amplitude} - N2_{amplitude})/2$. Each value in the table is always the arithmetic mean of two measured values for each stimulation and for each digital blur value. Values in this table marked NaN are non-detectable values from PR 15´, Blur 4 records.

PR 15´			PR 60´			MO TF2			MO TF4		
0 D	2 D	4 D	0 D	2 D	4 D	0 D	2 D	4 D	0 D	2 D	4 D
PR-Am						MO-Am					
6.3	3.9	NaN	7.1	6.7	6.6	2.8	1.9	1.6	5	3.5	2.7
3.9	3.8	1.3	6.1	5.5	3.2	0.8	1.5	2.3	2.1	1.9	1.3
9	7.7	1.4	5.4	7.1	6.9	2.3	2.2	1.7	2.6	2.3	2.4
4.4	3.9	0.6	3.9	4.3	3.4	5.3	4.4	1.7	4.1	2.9	2.3
7.1	3.7	NaN	8.5	6.8	5.7	3.6	1.7	3	3.6	2.7	3.2
7.9	1.8	NaN	7.1	6.6	6.9	1.9	0.9	0.9	1.2	1.5	2.1
6.6	3.2	-0.1	10	9.1	6.6	2.8	2.2	2.8	4.6	3.6	3.6
5.5	3.5	NaN	8.3	8.7	5.1	1.7	1.4	2.1	2.3	2.7	1.5
6	1.3	0.8	4.7	4.5	3.1	0.9	1.6	1	2.3	1.8	2.1
5.9	4.1	1.3	9	9.3	8.1	2.5	2	1.5	3.8	1.2	2.1
4.6	3.1	NaN	7.8	7.1	4.2	2.3	2.7	3	1.8	3.4	3.9
7.6	9.6	NaN	10.5	9.5	7.6	2.1	4.7	4.1	5	3.9	3.9
4	2	0.6	6.7	4.5	4.2	3.5	1.7	2.1	3.6	2.6	3.1

Table S3 – [ms/D] - peak time - the slopes of the regression lines for P100 positivity (PR VEP) and N2 negativity (MO VEP)

	subject	PR 15´	PR 60´	MO TF2	MO TF4
the slopes of the regression lines	S001	2.4	0.76	-2.725	-6.04
	S002	1.6	2.2	-1.005	4
	S003	10.35	2.1	-4.52	-5.04
	S004	13.125	2.675	1.56	2.6
	S005	8.25	1.025	-0.4	-1
	S006	1.2	1	-10.8	1.88
	S007	12.575	1.3	1.88	-0.4
	S008	3.45	-0.85	2.68	-0.24
	S009	15.55	0.45	-4.08	9.12
	S010	3.275	-0.8	-4.08	-8.76
	S011	1.05	0.075	-3.56	-8
	S012	1.35	-1.9	-3.52	0.6
	S013	0.125	1.125	2.2	-8.84

Table S4 – [μV/D] - interpeak amplitude - the slopes of the regression lines for PR-Am = ($P100_{amplitude}$ - $N75_{amplitude}$)/2 + ($P100_{amplitude}$ - $N145_{amplitude}$)/2 and MO-Am = ($P1_{amplitude}$ - $N2_{amplitude}$)/2 + ($P2_{amplitude}$ - $N2_{amplitude}$)/2.

	subject	PR 15´	PR 60´	MO TF2	MO TF4
the slopes of the regression lines	S001	-1.2055	-0.1328	-0.3022	-0.5671
	S002	-0.6639	-0.7076	0.3622	-0.1796
	S003	-1.8926	0.3779	-0.1593	-0.0352
	S004	-0.9515	-0.1211	-0.9107	-0.4476
	S005	-1.7042	-0.7002	-0.1265	-0.1232
	S006	-3.0622	-0.0369	-0.2491	0.2138
	S007	-1.6889	-0.8463	-0.0015	-0.2608
	S008	-1.004	-0.7929	0.108	-0.2012
	S009	-1.3027	-0.4009	0.025	-0.073
	S010	-1.1325	-0.2335	-0.2639	-0.4326
	S011	-0.74	-0.9074	0.173	0.512
	S012	0.9946	-0.7361	0.4863	-0.2603
	S013	-0.8451	-0.6368	-0.3581	-0.1179

Motion onset VEPs can see through the blur.

D. Kordek[1], L. K. Young[2], P. Voda[1], J. Kremlacek[1]

[1]Department of Medical Biophysics. Faculty of Medicine in Hradec Kralove. Charles University. Hradec Kralove. Czech Republic

[2]Biosciences Institute. Newcastle University. Newcastle. UK

List of figures in digital_blur_stimuli.zip

Checkerboard_defocus_000_size_015.png - checkerboard with 15' square size, digital blur corresponding to equivalent defocus 0 D (Blur 0)

Checkerboard_defocus_200_size_015.png - checkerboard with 15' square size, digital blur corresponding to equivalent defocus 2 D (Blur 2)

Checkerboard_defocus_400_size_015.png - checkerboard with 15' square size, digital blur corresponding to 4 D equivalent defocus (Blur 4)

Checkerboard_defocus_000_size_060.png - checkerboard with square size 60', digital blur corresponding to equivalent defocus 0 D (Blur 0)

Checkerboard_defocus_200_size_060.png - checkerboard with square size 60', digital blur corresponding to equivalent defocus 2 D (Blur 2)

Checkerboard_defocus_400_size_060.png - checkerboard with square size 60', digital blur corresponding to equivalent defocus 4 D (Blur 4)

The resolution of all images is 1024 × 768 px.

Motion onset VEPs can see through the blur.

D. Kordek[1], L. K. Young[2], P. Voda[1], J. Kremlacek[1]

[1]Department of Medical Biophysics. Faculty of Medicine in Hradec Kralove. Charles University. Hradec Kralove. Czech Republic

[2]Biosciences Institute. Newcastle University. Newcastle. UK

Description of VEP_raw_data.zip content

The VEP data were exported from TruTrace (ver 7.11, Deymed Ltd. Czech Republic) as a segmented ASCII text file. The file contains numbers separated by a tabulator. Every file includes averaged Visual Evoked Potential (VEP) traces recorded from 6 unipolar channels (Ol, Oz, Or, Fz, Cz, Pz – reference A2) and one bipolar channel (Oz-Fz) for a single subject and one condition (see the table below). Data were sampled at 6250 Hz. Recording began in 2061 and ended with 6248 samples after the trigger.

Here's a breakdown of the file structure:

1. **Header Information**: The file begins with some general settings and parameters for the data:
   - `reserved fields`: Two zeros.
   - `bits per unit`: Max size of the packet – *value is not relevant in our files*.
   - `sampling frequency`: The rate at which the data was collected.
   - `notch filter frequency`: The frequency used for notch filtering to remove power line interference.
   - `first sample position`: The position in the data where the first sample is located – *value is not relevant in our files*.
   - `signal start sample` and `signal end sample`: The range of the data that contains the signal of interest.
   - `channels count`: The number of channels used in the data collection.
2. **Channel Information**: For each channel, the file contains:
   - `trigger count`: The number of triggers in the data.
   - `examination type`: The type of examination performer – *value is not relevant in our files*.
   - `side`: The side of the stimulation/recording (1=right, 2=left) – *value is not relevant in our files*.
   - `channel position`: The position of the channel on the scalp – *value is not relevant in our files*.
   - `index of channel in set`: The index of the channel in the set of all channels.
   - **Marker Information**: For each channel, there are several markers, each with:
     - `Marker index`: The index of the marker – *value is not relevant in our files*.
     - `Marker latency`: The latency of the marker – *value is not relevant in our files*.
   - **Reserved Fields**:

        - `reserved fields`: Twelve zeros.
    - **Samples**: The actual VEP data is stored as a series of samples. For each for each sample, there is a `sample value` that represents the recorded voltage at that point in time.

subject	stimulation	blur	link
S001	rev60	0	SourceBlurVEP-S001_VEP_2023-03-23_08-35-38.txt
S001	rev60	2	SourceBlurVEP-S001_VEP_2023-03-23_08-38-33.txt
S001	rev60	4	SourceBlurVEP-S001_VEP_2023-03-23_08-41-17.txt
S001	rev15	0	SourceBlurVEP-S001_VEP_2023-03-23_08-42-52.txt
S001	rev15	2	SourceBlurVEP-S001_VEP_2023-03-23_08-44-32.txt
S001	rev15	4	SourceBlurVEP-S001_VEP_2023-03-23_08-46-01.txt
S002	rev60	0	SourceBlurVEP-S002_VEP_2023-03-24_11-01-40.txt
S002	rev60	2	SourceBlurVEP-S002_VEP_2023-03-24_10-58-48.txt
S002	rev60	4	SourceBlurVEP-S002_VEP_2023-03-24_10-57-01.txt
S002	rev15	0	SourceBlurVEP-S002_VEP_2023-03-24_10-55-04.txt
S002	rev15	2	SourceBlurVEP-S002_VEP_2023-03-24_10-52-51.txt
S002	rev15	4	SourceBlurVEP-S002_VEP_2023-03-24_10-50-34.txt
S003	rev60	0	SourceBlurVEP-S003_VEP_2023-03-29_10-42-37.txt
S003	rev60	2	SourceBlurVEP-S003_VEP_2023-03-29_10-40-03.txt
S003	rev60	4	SourceBlurVEP-S003_VEP_2023-03-29_10-44-53.txt
S003	rev15	0	SourceBlurVEP-S003_VEP_2023-03-29_10-57-27.txt
S003	rev15	2	SourceBlurVEP-S003_VEP_2023-03-29_10-55-45.txt
S003	rev15	4	SourceBlurVEP-S003_VEP_2023-03-29_10-59-14.txt
S004	rev60	0	SourceBlurVEP-S004_VEP_2023-03-30_07-55-10.txt
S004	rev60	2	SourceBlurVEP-S004_VEP_2023-03-30_07-56-49.txt
S004	rev60	4	SourceBlurVEP-S004_VEP_2023-03-30_07-58-44.txt
S004	rev15	0	SourceBlurVEP-S004_VEP_2023-03-30_08-09-33.txt
S004	rev15	2	SourceBlurVEP-S004_VEP_2023-03-30_08-11-10.txt
S004	rev15	4	SourceBlurVEP-S004_VEP_2023-03-30_08-18-14.txt
S005	rev60	0	SourceBlurVEP-S005_VEP_2023-03-31_11-38-03.txt
S005	rev60	2	SourceBlurVEP-S005_VEP_2023-03-31_11-35-24.txt
S005	rev60	4	SourceBlurVEP-S005_VEP_2023-03-31_11-33-33.txt
S005	rev15	0	SourceBlurVEP-S005_VEP_2023-03-31_11-31-41.txt
S005	rev15	2	SourceBlurVEP-S005_VEP_2023-03-31_11-29-50.txt
S005	rev15	4	SourceBlurVEP-S005_VEP_2023-03-31_11-28-05.txt
S006	rev60	0	SourceBlurVEP-S006_VEP_2023-04-03_09-16-37.txt
S006	rev60	2	SourceBlurVEP-S006_VEP_2023-04-03_09-21-30.txt
S006	rev60	4	SourceBlurVEP-S006_VEP_2023-04-03_09-14-36.txt
S006	rev15	0	SourceBlurVEP-S006_VEP_2023-04-03_09-10-42.txt
S006	rev15	2	SourceBlurVEP-S006_VEP_2023-04-03_09-12-40.txt
S006	rev15	4	SourceBlurVEP-S006_VEP_2023-04-03_09-08-46.txt
S007	rev60	0	SourceBlurVEP-S007_VEP_2023-04-04_09-47-19.txt
S007	rev60	2	SourceBlurVEP-S007_VEP_2023-04-04_09-49-17.txt
S007	rev60	4	SourceBlurVEP-S007_VEP_2023-04-04_09-52-23.txt

S007	rev15	0	SourceBlurVEP-S007_VEP_2023-04-04_09-22-47.txt
S007	rev15	2	SourceBlurVEP-S007_VEP_2023-04-04_09-24-36.txt
S007	rev15	4	SourceBlurVEP-S007_VEP_2023-04-04_09-26-34.txt
S008	rev60	0	SourceBlurVEP-S008_VEP_2023-04-05_14-49-18.txt
S008	rev60	2	SourceBlurVEP-S008_VEP_2023-04-05_14-46-29.txt
S008	rev60	4	SourceBlurVEP-S008_VEP_2023-04-05_14-44-36.txt
S008	rev15	0	SourceBlurVEP-S008_VEP_2023-04-05_14-55-27.txt
S008	rev15	2	SourceBlurVEP-S008_VEP_2023-04-05_14-53-22.txt
S008	rev15	4	SourceBlurVEP-S008_VEP_2023-04-05_14-51-20.txt
S009	rev60	0	SourceBlurVEP-S009_VEP_2023-04-12_10-35-21.txt
S009	rev60	2	SourceBlurVEP-S009_VEP_2023-04-12_10-37-07.txt
S009	rev60	4	SourceBlurVEP-S009_VEP_2023-04-12_10-39-01.txt
S009	rev15	0	SourceBlurVEP-S009_VEP_2023-04-12_10-40-51.txt
S009	rev15	2	SourceBlurVEP-S009_VEP_2023-04-12_10-42-45.txt
S009	rev15	4	SourceBlurVEP-S009_VEP_2023-04-12_10-44-50.txt
S010	rev60	0	SourcBlurVEP-S010_VEP_2023-04-14_14-49-58.txt
S010	rev60	2	SourcBlurVEP-S010_VEP_2023-04-14_14-47-57.txt
S010	rev60	4	SourcBlurVEP-S010_VEP_2023-04-14_14-52-26.txt
S010	rev15	0	SourcBlurVEP-S010_VEP_2023-04-14_14-32-52.txt
S010	rev15	2	SourcBlurVEP-S010_VEP_2023-04-14_14-31-06.txt
S010	rev15	4	SourcBlurVEP-S010_VEP_2023-04-14_14-35-29.txt
S011	rev60	0	SourceBlurVEP-S011_VEP_2023-04-17_14-14-56.txt
S011	rev60	2	SourceBlurVEP-S011_VEP_2023-04-17_14-12-14.txt
S011	rev60	4	SourceBlurVEP-S011_VEP_2023-04-17_14-09-07.txt
S011	rev15	0	SourceBlurVEP-S011_VEP_2023-04-17_14-06-52.txt
S011	rev15	2	SourceBlurVEP-S011_VEP_2023-04-17_14-05-05.txt
S011	rev15	4	SourceBlurVEP-S011_VEP_2023-04-17_14-02-03.txt
S012	rev60	0	SorceBlurVEP-S012_VEP_2023-04-18_15-06-09.txt
S012	rev60	2	SorceBlurVEP-S012_VEP_2023-04-18_15-01-37.txt
S012	rev60	4	SorceBlurVEP-S012_VEP_2023-04-18_15-03-28.txt
S012	rev15	0	SorceBlurVEP-S012_VEP_2023-04-18_15-12-01.txt
S012	rev15	2	SorceBlurVEP-S012_VEP_2023-04-18_15-08-07.txt
S012	rev15	4	SorceBlurVEP-S012_VEP_2023-04-18_15-10-05.txt
S013	rev60	0	SorceBlurVEP-S013_VEP_2023-04-18_16-00-00.txt
S013	rev60	2	SorceBlurVEP-S013_VEP_2023-04-18_15-58-08.txt
S013	rev60	4	SorceBlurVEP-S013_VEP_2023-04-18_16-02-21.txt
S013	rev15	0	SorceBlurVEP-S013_VEP_2023-04-18_15-44-13.txt
S013	rev15	2	SorceBlurVEP-S013_VEP_2023-04-18_15-42-02.txt
S013	rev15	4	SorceBlurVEP-S013_VEP_2023-04-18_15-46-39.txt
S001	TF2	0	SourceBlurVEP-S001_VEP_2023-03-23_08-59-55.txt
S001	TF2	2	SourceBlurVEP-S001_VEP_2023-03-23_09-03-16.txt
S001	TF2	4	SourceBlurVEP-S001_VEP_2023-03-23_10-37-53.txt
S001	TF4	0	SourceBlurVEP-S001_VEP_2023-03-23_08-49-44.txt
S001	TF4	2	SourceBlurVEP-S001_VEP_2023-03-23_08-53-21.txt
S001	TF4	4	SourceBlurVEP-S001_VEP_2023-03-23_08-57-00.txt

S002	TF2	0	SourceBlurVEP-S002_VEP_2023-03-24_10-48-46.txt
S002	TF2	2	SourceBlurVEP-S002_VEP_2023-03-24_10-45-08.txt
S002	TF2	4	SourceBlurVEP-S002_VEP_2023-03-24_10-42-43.txt
S002	TF4	0	SourceBlurVEP-S002_VEP_2023-03-24_10-38-42.txt
S002	TF4	2	SourceBlurVEP-S002_VEP_2023-03-24_10-36-22.txt
S002	TF4	4	SourceBlurVEP-S002_VEP_2023-03-24_10-32-02.txt
S003	TF2	0	SourceBlurVEP-S003_VEP_2023-03-29_11-05-26.txt
S003	TF2	2	SourceBlurVEP-S003_VEP_2023-03-29_11-02-06.txt
S003	TF2	4	SourceBlurVEP-S003_VEP_2023-03-29_11-09-33.txt
S003	TF4	0	SourceBlurVEP-S003_VEP_2023-03-29_10-50-45.txt
S003	TF4	2	SourceBlurVEP-S003_VEP_2023-03-29_10-47-48.txt
S003	TF4	4	SourceBlurVEP-S003_VEP_2023-03-29_10-54-02.txt
S004	TF2	0	SourceBlurVEP-S004_VEP_2023-03-30_07-47-34.txt
S004	TF2	2	SourceBlurVEP-S004_VEP_2023-03-30_07-50-28.txt
S004	TF2	4	SourceBlurVEP-S004_VEP_2023-03-30_07-53-25.txt
S004	TF4	0	SourceBlurVEP-S004_VEP_2023-03-30_08-01-31.txt
S004	TF4	2	SourceBlurVEP-S004_VEP_2023-03-30_08-04-20.txt
S004	TF4	4	SourceBlurVEP-S004_VEP_2023-03-30_08-07-23.txt
S005	TF2	0	SourceBlurVEP-S005_VEP_2023-03-31_11-57-16.txt
S005	TF2	2	SourceBlurVEP-S005_VEP_2023-03-31_11-54-42.txt
S005	TF2	4	SourceBlurVEP-S005_VEP_2023-03-31_11-51-17.txt
S005	TF4	0	SourceBlurVEP-S005_VEP_2023-03-31_11-48-03.txt
S005	TF4	2	SourceBlurVEP-S005_VEP_2023-03-31_11-44-38.txt
S005	TF4	4	SourceBlurVEP-S005_VEP_2023-03-31_11-41-15.txt
S006	TF2	0	SourceBlurVEP-S006_VEP_2023-04-03_08-53-30.txt
S006	TF2	2	SourceBlurVEP-S006_VEP_2023-04-03_08-56-49.txt
S006	TF2	4	SourceBlurVEP-S006_VEP_2023-04-03_08-50-22.txt
S006	TF4	0	SourceBlurVEP-S006_VEP_2023-04-03_09-03-15.txt
S006	TF4	2	SourceBlurVEP-S006_VEP_2023-04-03_09-06-48.txt
S006	TF4	4	SourceBlurVEP-S006_VEP_2023-04-03_09-00-03.txt
S007	TF2	0	SourceBlurVEP-S007_VEP_2023-04-04_09-39-04.txt
S007	TF2	2	SourceBlurVEP-S007_VEP_2023-04-04_09-42-08.txt
S007	TF2	4	SourceBlurVEP-S007_VEP_2023-04-04_09-45-19.txt
S007	TF4	0	SourceBlurVEP-S007_VEP_2023-04-04_09-29-35.txt
S007	TF4	2	SourceBlurVEP-S007_VEP_2023-04-04_09-32-48.txt
S007	TF4	4	SourceBlurVEP-S007_VEP_2023-04-04_09-35-52.txt
S008	TF2	0	SourceBlurVEP-S008_VEP_2023-04-05_15-12-59.txt
S008	TF2	2	SourceBlurVEP-S008_VEP_2023-04-05_15-03-35.txt
S008	TF2	4	SourceBlurVEP-S008_VEP_2023-04-05_14-58-47.txt
S008	TF4	0	SourceBlurVEP-S008_VEP_2023-04-05_14-42-24.txt
S008	TF4	2	SourceBlurVEP-S008_VEP_2023-04-05_14-39-03.txt
S008	TF4	4	SourceBlurVEP-S008_VEP_2023-04-05_14-35-53.txt
S009	TF2	0	SourceBlurVEP-S009_VEP_2023-04-12_10-57-02.txt
S009	TF2	2	SourceBlurVEP-S009_VEP_2023-04-12_11-00-02.txt
S009	TF2	4	SourceBlurVEP-S009_VEP_2023-04-12_11-03-47.txt

S009	TF4	0	SourceBlurVEP-S009_VEP_2023-04-12_10-47-56.txt
S009	TF4	2	SourceBlurVEP-S009_VEP_2023-04-12_10-50-51.txt
S009	TF4	4	SourceBlurVEP-S009_VEP_2023-04-12_10-53-54.txt
S010	TF2	0	SourcBlurVEP-S010_VEP_2023-04-14_14-42-08.txt
S010	TF2	2	SourcBlurVEP-S010_VEP_2023-04-14_14-38-56.txt
S010	TF2	4	SourcBlurVEP-S010_VEP_2023-04-14_14-45-23.txt
S010	TF4	0	SourcBlurVEP-S010_VEP_2023-04-14_14-58-48.txt
S010	TF4	2	SourcBlurVEP-S010_VEP_2023-04-14_14-55-42.txt
S010	TF4	4	SourcBlurVEP-S010_VEP_2023-04-14_15-03-04.txt
S011	TF2	0	SourceBlurVEP-S011_VEP_2023-04-17_13-50-33.txt
S011	TF2	2	SourceBlurVEP-S011_VEP_2023-04-17_13-47-06.txt
S011	TF2	4	SourceBlurVEP-S011_VEP_2023-04-17_13-42-31.txt
S011	TF4	0	SourceBlurVEP-S011_VEP_2023-04-17_13-59-40.txt
S011	TF4	2	SourceBlurVEP-S011_VEP_2023-04-17_13-56-48.txt
S011	TF4	4	SourceBlurVEP-S011_VEP_2023-04-17_13-53-44.txt
S012	TF2	0	SorceBlurVEP-S012_VEP_2023-04-18_14-59-44.txt
S012	TF2	2	SorceBlurVEP-S012_VEP_2023-04-18_14-53-16.txt
S012	TF2	4	SorceBlurVEP-S012_VEP_2023-04-18_14-56-34.txt
S012	TF4	0	SorceBlurVEP-S012_VEP_2023-04-18_15-22-42.txt
S012	TF4	2	SorceBlurVEP-S012_VEP_2023-04-18_15-15-05.txt
S012	TF4	4	SorceBlurVEP-S012_VEP_2023-04-18_15-18-41.txt
S013	TF2	0	SorceBlurVEP-S013_VEP_2023-04-18_16-10-22.txt
S013	TF2	2	SorceBlurVEP-S013_VEP_2023-04-18_16-06-33.txt
S013	TF2	4	SorceBlurVEP-S013_VEP_2023-04-18_16-44-25.txt
S013	TF4	0	SorceBlurVEP-S013_VEP_2023-04-18_15-52-49.txt
S013	TF4	2	SorceBlurVEP-S013_VEP_2023-04-18_15-49-45.txt
S013	TF4	4	SorceBlurVEP-S013_VEP_2023-04-18_15-56-15.txt
